# Metabolic network reconstruction and phenome analysis of the industrial microbe, *Escherichia coli* BL21(DE3)

**DOI:** 10.1371/journal.pone.0204375

**Published:** 2018-09-21

**Authors:** Hanseol Kim, Sinyeon Kim, Sung Ho Yoon

**Affiliations:** Department of Bioscience and Biotechnology, Konkuk University, Seoul, Republic of Korea; Universite Paris-Sud, FRANCE

## Abstract

*Escherichia coli* BL21(DE3) is an industrial model microbe for the mass-production of bioproducts such as biofuels, biorefineries, and recombinant proteins. However, despite its important role in scientific research and biotechnological applications, a high-quality metabolic network model for metabolic engineering is yet to be developed. Here, we present the comprehensive metabolic network model of *E*. *coli* BL21(DE3), named iHK1487, based on the latest genome reannotation and phenome analysis. The metabolic model consists of 1,164 unique metabolites, 2,701 metabolic reactions, and 1,487 genes. The model was validated and improved by comparing the simulation results with phenome data from phenotype microarray tests. Previous transcriptome profile data was incorporated during model reconstruction, and flux prediction was simulated using the model. iHK1487 was simulated to explore the metabolic features of BL21(DE3) such as broad spectrum amino acid utilization and enhanced flux through the upper glycolytic pathway and TCA cycle. iHK1487 will contribute to systematic understanding of cellular physiology and metabolism of *E*. *coli* BL21(DE3) and highlight its biotechnological applications.

## Introduction

Modeling and simulation of metabolic networks are well-established computational tools for myriad applications such as designing of microbial cell factories, model-driven discovery, and phenotype prediction [1–4]. The recent development of sequencing technology and accumulation of biochemical and enzymatic data has facilitated the reconstruction of genome-wide metabolic networks in diverse organisms [5,6]. However, reconstruction of a comprehensive and accurate metabolic network model is a time- and labor-consuming task. To tackle this problem, a protocol for genome-scale metabolic reconstruction was suggested [7]. Several methods have been developed to support model reconstruction in a (semi-) automatic manner [8–10]. Basically, these methods convert genome annotation into a genome-scale metabolic model. Thus, the automated approach generates a draft reconstruction, which can be easily falsified by (i) incomplete and erroneous genome annotation, (ii) inconsistent naming of metabolites and reactions among different data sources, and (iii) conflicting information on reversibility and activity of metabolic reactions [11,12]. Thus, high quality metabolic network reconstruction requires extensive manual curation based on expert knowledge and literature survey.

The K-12 and B strains of *Escherichia coli* and their derivatives have been widely used and have had enormous impact on basic biology, medicine, and biotechnology. K-12 strains have been the primary choice for genetic studies, and hence, extensive genetic, metabolic, and omic studies of *E*. *coli* have been performed using K-12 or its derivatives. After the release of the complete genome sequence of *E*. *coli* K-12 MG1655 in 1997 [13], global efforts have been dedicated to generate a functional update of the K-12 genome [14–17], as a result of which, this strain has the most comprehensive and curated metabolic network model [1,18].

Derivatives of *E*. *coli* B, especially BL21(DE3) [19], have been widely used for the overproduction of recombinant proteins, biofuels, and biorefineries owing to several favorable features such as faster growth in minimal media, lower acetate production, higher expression levels of recombinant proteins, and less degradation of such proteins during purification [20,21]. Despite their importance in biotechnology, omics and systems biology studies on B strains are limited. In 2009, the entire genome sequences of two *E*. *coli* B strains, BL21(DE3) and REL606, were first completed and annotated [22,23]. The genome information has enabled multi-omics analysis and genome-scale metabolic network of *E*. *coli* REL606 [3]. Recently, the BL21(DE3) genome was re-annotated and the transcriptome structure of the strain was determined [24].

*E*. *coli* BL21(DE3) is one of the most widely used industrial workhorses, especially for the overproduction of recombinant proteins [21]. BL21(DE3) was derived from BL21 by integrating the DE3 prophage in the lambda attachment site of BL21 to use phage T7 RNA polymerase for recombinant protein production [19]. Compared to the highly curated and fine-tuned metabolic network model of *E*. *coli* K-12 MG1655 [25], development of the metabolic network of *E*. *coli* BL21(DE3) is in early stages. The first metabolic model of *E*. *coli* BL21, iECD_1391 [26,27], was reconstructed in a semi-automatic manner using the ModelSEED pipeline [8]. Considering the recent genome reannotation and biotechnological importance of BL21(DE3), its metabolic network model has to be reconstructed via expert manual curation.

In this study, we reconstructed a comprehensive and highly-curated metabolic network model of *E*. *coli* BL21(DE3) based on the recent genome reannotation and phenome analysis. To exploit the well-developed metabolic model of K-12 MG1655 [25], we highlighted similarities and differences between the genomes of BL21(DE3) and K-12 MG1655. Phenotype microarray (PM) test was performed to validate and corroborate the network model. Finally, the reconstructed model was simulated to explore the metabolic features of BL21(DE3).

## Materials and methods

### Metabolic network reconstruction

The metabolic network model of *E*. *coli* BL21(DE3) was reconstructed based on recent genome annotation of *E*. *coli* BL21(DE3) [24] and metabolic model of *E*. *coli* K-12 MG1655 (iML1515) [25]. The K-12 iML1515 consists of 1,515 genes, 2,719 metabolic reactions, and 1,192 unique metabolites, which was downloaded from the BiGG database [6] in the SBML file format. Genes present both in the BL21(DE3) and K-12 genomes were retrieved from the publication on genome reannotation of BL21(DE3) [24], and their corresponding metabolic reactions were obtained from iML1515. Reactions that depend only on K-12-specific genes and pseudogenes were excluded from iML1515. Genes unique to BL21(DE3) and their corresponding reactions were retrieved from the recent genome reannotation [24] and MetaCyc database [28], respectively.

### Phenotype microarray test

*E*. *coli* BL21(DE3) [19] was provided by F. William Studier, Brookhaven National Laboratory, and *E*. *coli* K-12 MG1655 was purchased from American Type Culture Collection (ATCC). PM tests on BL21(DE3) and K-12 strains were performed as described previously [3]. The PM plates (Biolog Inc., Hayward, CA, USA) consisted of 20 96-well plates containing different sources of carbon (PM1 and PM2), nitrogen (PM3), phosphorus, and sulfur (PM4), auxotrophic supplements (PM5 to PM8), or salt (PM9). The PM10 plate was used to test pH stress, whereas PM11 to PM20 plates contained inhibitory compounds such as antibiotics, antimetabolites, and other inhibitors. Cells were grown overnight at 37°C on Biolog universal growth (BUG) + B agar plate. Colonies were picked from the agar surface and suspended in inoculating fluid (IF) containing the indicator dye tetrazolium violet. The IF-0 media was used for plates PM1 to PM8 and IF-10 for plates PM9 to PM20. Sodium succinate or pyruvate was added with ferric citrate to the inoculation solution of plates PM3 to PM8. All PM plates were inoculated with cell suspensions at 100 μl/well and incubated in an OmniLog incubator (Biolog Inc.) at 37°C for 30 or 48 hours. Five independent PM tests were performed on BL21(DE3) by varying the carbon source for the plates PM3 to PM8 (sodium pyruvate for three replicates and sodium succinate for two replicates). One PM test using sodium pyruvate as the carbon source for the plates PM3 to PM8 was performed on K-12 MG1655. Four independent PM tests on K-12 and five PM tests on B REL606, which used sodium succinate as the carbon source for the plates PM3 to PM8, were retrieved from our previous study [3]. The PM data was analyzed using the opm R package [29], and the bacterial cell growth in each well was classified into negative, weak (or ambiguous), and positive growths.

### Flux balance analysis

Flux balance analysis (FBA) [30] of the reconstructed metabolic model of BL21(DE3) was performed to simulate cell growth using Constraints-Based Reconstruction and Analysis for python (COBRApy) [31]. The maximum growth rate from the core biomass equation [25] was used as the objective function. The upper limit of uptake rates of glucose and oxygen were set as 10 mmol gDCW^-1^ h^-1^ and 18.5 mmol gDCW^-1^ h^-1^, respectively, unless otherwise mentioned.

For simulation of cell growth on PM plates for testing carbon source utilization (PM1 and PM2), the maximum uptake rate of each carbon source was set at 10 mmol gDCW^-1^ h^-1^. The composition of minimal media IF-0 used in PM1 and PM2 (136.1 mM chloride, 104.5 mM Na^+^, 30 mM triethanolamine, 5 mM ammonium, 2 mM phosphate, 1 mM K^+^, 0.25 mM sulfate, 0.05 mM Mg^2+^, and 0.001 mM Fe^3+^) was retrieved from the BioCyc web site [32] and was used as the upper limit of the uptake rate of the corresponding component. A substrate was considered to be not metabolized if the growth rate was less than 5% of the growth objective value calculated for the wild type strain.

To identify the reactions that are essential for cell growth, the maximum growth rate was simulated when each reaction was removed from the reconstructed metabolic model using the function “find_essential_genes” in COBRApy [31]. An unrestrained supply of all the substrates was assumed. A reaction was considered essential if its removal from the model reduced growth rate below the default threshold of 5% of the growth objective value calculated for the wild type strain.

## Results

### Overview of the metabolic model reconstruction

We reconstructed a genome-scale metabolic network model of BL21(DE3) by comparing the genomes of BL21(DE3) and K-12 (Table 1). The genomes of BL21(DE3) and K-12 MG1655 are similar in terms of gene contents and sequence identity [23,24,33]. Thus, we utilized the metabolic model of K-2 MG1655 (iML1515) [25], which is the most comprehensive and accurate model in microorganisms, for reconstructing the BL21(DE3) metabolic model, starting with the identification of the gene set commonly present in BL21(DE3) and K-12 MG1655. The 1,452 metabolism-related genes (96%) in the K-12 metabolic model (iML1515) were also detected in the BL21(DE3) genome, leading to the identification of 2,637 metabolic reactions (97% of iML1515) shared by the two genomes.

**Table 1:** Comparison of the metabolic network models of *E*. *coli* BL21(DE3).

Metabolic model	iECD_1391	iHK1487
**Genes**	1355	1487
**Reactions**	2747	2701
Metabolic reactions	1766	1650
Transport reactions	650	705
Exchange reactions	331	347
**Gene-reaction association**	2747	2701
Gene-associated reactions	2209	2205
Not gene-associated reactions	502	460
Spontaneous reactions	36	37
**Metabolites**	1924	1877
Cytoplasmic	1105	1062
Periplasmic	478	469
Extracellular	341	346
**Unique metabolites**	1225	1164

Thirty-nine metabolic genes were present only in the BL21(DE3) genome, and their associated metabolic reactions and metabolites were identified using exhaustive searches in biochemical literature and the MetaCyc database [28], leading to the addition of 27 metabolites and 51 metabolic reactions, including 11 exchange reactions into the metabolic model (S1 and S2 Tables). Each of the 51 added reactions was verified to be connected properly to the preexisting network by performing flux variability analysis [34]. Among the 39 added genes, 34 genes associated with 25 reactions were expressed in more than one condition among the five culture/growth conditions of our transcriptome analysis of BL21(DE3) [24] (S3 Table). Although the remaining five genes were not expressed in any of the five conditions, they were added to the model because their product annotations were updated to be functional from the previous genome reannotation [24]. We did not add genes which were annotated to have putative or hypothetical functions without any transcriptional evidence. For example, ECD_04073, encoding the putative acetyl-CoA:acetoacetyl-CoA transferase, is absent in the K-12 genome and is highly similar to the gene encoding propanoyl-CoA transferase of *Megasphaera elsdenii* (6 × 10^−141^ e-value, 98% coverage, and 46% amino acid identity); however, it was not expressed in any of the transcriptome data set.

From the reaction list of K-12 MG1655 model (iML1515) [25], 70 reactions specific to the K-12 genome were excluded, such as those involved in D-galactose utilization and phenylacetic acid degradation. Sixty-four genes and their related reactions in iML1515 were removed, as those genes were absent or annotated as pseudogenes in the BL21(DE3) genome (S4 Table). The draft model was further revised based on the PM results (see below). The resulting metabolic network of BL21(DE3), referred to as iHK1487, consisted of 1,487 genes, 1,164 unique metabolites, and 2,701 reactions. The model can be downloaded in Excel (S5 Table) and SBML format (S1 File), which are input to the COBRA softwares [31,35].

### Comparative analysis of BL21(DE3) and K-12 genomes

**Fig 1:**
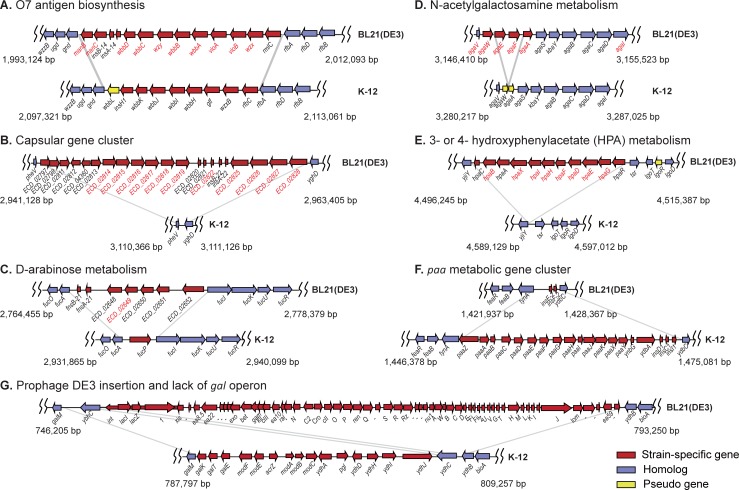
Differential metabolic gene clusters in the genomes of *E*. *coli* BL21(DE3) and *E*. *coli* K-12 MG1655. Presence of gene clusters for O7 antigen biosynthesis (**A**), capsular gene cluster (**B**), D-arabinose metabolism (**C**), N-acetylgalactosamine metabolism (**D**), and 3- or 4- hydroxyphenylacetate metabolism (**E**). Absence of gene clusters for phenyl acetic acid utilization (**F**). Prophage DE3 insertion and lack of gene clusters for galactose metabolism and molybdate ABC transporters (**G**). Gene names in red are for BL21(DE3) genes, the reactions of which were included in the BL21(DE3) metabolic model.

Comparison of BL21(DE3) and K-12 genomes identified the genomic regions of BL21(DE3) that differ from those of K-12 MG1655 (Fig 1). The 51 metabolic reactions and 27 metabolites detected only in the BL21(DE3) genome were associated with O7 antigen biosynthesis [36], capsular polysaccharide biosynthesis [37,38], and 3- or 4-hydroxyphenylacetate (HPA), D-arabinose, and N-acetylgalactosamine metabolism.

BL21(DE3) can synthesize the O7 antigen due to the presence and expression of the intact gene cluster for O7 antigen biosynthesis (*rfb* genes) (Fig 1A) [24]. However, K-12 cannot synthesize the O16 antigen because of disruption of *wbbL* by insertion sequence (IS) element integration [23,39]. The BL21(DE3) genome contains a group II capsular gene (*kps*) cluster (ECD_02813 to ECD_02828) which was presumably acquired by horizontal gene transfer in the K-12 *pheV* locus (Fig 1B) [38]. The metabolic genes of *Haemophilus influenzae* for capsular polysaccharide biosynthesis [37] were homologous to the 11 genes in the *kps* cluster of BL21(DE3) [24], and their metabolic reactions were added to the metabolic model. ECD_02649, encoding the L-ribulokinase AraB-like protein, is located between *fucA* and *fucI* of BL21(DE3) (Fig 1C), and its associated reaction for D-arabinose catabolism (RBK_D1) were retrieved from the REL606 metabolic model [3]. BL21(DE3) harbors an intact *aga* operon for N-acetylgalactosamine metabolism (Fig 1D). Different gene clusters for the catabolism of aromatic compounds were present in the two strains, namely, the *hpa* cluster for 3- and 4-hydroxy phenyl acetic acid (HPA) catabolism in the BL21(DE3) genome (Fig 1E) and the *paa* cluster for phenyl acetic acid (PAA) utilization in the K-12 genome (Fig 1F). Although the BL21(DE3) genome has two large gene clusters for the DE3 prophage (Fig 1G) and additional type II secretion system, metabolic genes were not present in these clusters.

Interestingly, a large gene cluster was missing near the DE3 insertion site in the BL21(DE3) genome, which led to the absence of the *gal* operon (*galETKM*), molybdate ABC transporter genes (*modF* and *modABC*), and *pgl* (encoding 6-phosphogluconolactonase) in the oxidative pentose phosphate pathway of BL21(DE3) (Fig 1G). *pgl* is present both in B REL606 and K-12 genomes [3], but is absent in the BL21 genome [27]. This might imply that lack of *pgl* is not a B lineage-specific characteristic. BL21(DE3) appears to have reduced capacity for uptake of cobalamins such as vitamin B_12_ due to a nonsense mutation in *btuB*. Repression of BtuB synthesis resulted in resistance of *E*. *coli* to colicins [40].

### Identification of BL21(DE3)-specific metabolic regulation

To identify the BL21 specific regulation, the regulatory influences of transcription factors (TFs) on metabolic gene(s) were retrieved from the previous publication [25]. We also performed literature and database searches for additional regulatory influences. If a given TF is frameshifted or deactivated in BL21(DE3), we turned off its influenced metabolic reaction(s). Next, we checked whether the turned-off metabolic genes were not expressed using our pervious transcriptome profile data [24]. Some of the regulation were verified by PM data.

We deactivated 11 metabolic reactions (S1 Table) by setting the fluxes associated with BL21(DE3)-specific regulation to zero. Two regulatory genes, *IgoR* (encoding the putative regulator of L-galactonate metabolism) (Fig 1E) and *dcuS* (encoding C_4_-dicarboxylate-sensing histidine kinase), are frameshifted in BL21(DE3), whereas they are intact in K-12. The *IgoR* deletion mutant did not grow on L-galactonate [41] and our PM data showed that BL21(DE3) did not grow on L-galactonate. Thus, we turned off one reaction catalyzed by L-galactonate oxidoreductase and two L-galactonate transport reactions. DcuS is a sensor kinase of the two-component system DcuSR for the utilization of C_4_-dicarboxylates such as aspartate, fumarate, malate, and succinate [42]. DucS is the positive regulator of the fumarate/succinate antiporter (DcuB) and aerobic C_4_-dicarboxylate carrier (DctA) [42,43]. Our PM data also showed that among the C_4_-dicarboxylates, BL21(DE3) showed growth defects in using D-tartaric acid and D-malic acid as a carbon source (S6 Table). Hence, we deactivated eight transport reactions mediated by DctA and DcuB.

### Generation of biomass equation

The biomass equation of iML1515 for K-12 model [25] was modified in iHK1487. In the iML1515 biomass equation, K-12 specific factors are GC content, composition of metal cofactors (MoO_4_^-2^, Cu^+2^, Mn^+2^, Zn^+2^, Ni^+2^, and Co^+2^) [44], and growth-associated maintenance energy (GAM), while all the remaining compositions came from B/r [18,45,46]. B/r and BL21 shares the same ancestor of *E*. *coli* B [22].

We determined GAM and non-growth-associated maintenance energy (NGAM) which represent energy costs for cell maintenance and cell growth, respectively [7]. Specific growth rate versus glucose uptake rate, which were obtained from glucose-limited chemostat cultures of *E*. *coli* B/r growing aerobically on minimal medium [47], was plotted to calculate NGAM of 5.17 mmol ATP gDCW^-1^ h^-1^ from the y-intercept (S1 Fig). FBA was performed varying GAM in the biomass equation to find GAM leading to the closest fit of the experimental plot (70.12 mmol ATP gDCW^-1^). These values are lower than those from K-12 iML1515 (6.86 of NGAM and 75.55 of GAM), probably due to the nonmotility of B strains [21]. The influence of GAM and NGAM on the predicted rates of cell growth, oxygen uptake, and acetate production was investigated (S2 Fig). Under the simulation condition of aerobic growth with high glucose uptake rate (10 mmol gDCW^-1^ h^-1^), we varied values of GAM (from 20 to 100 mmol ATP gDCW^-1^) and NGAM (0 to10 mmol ATP gDCW^-1^ h^-1^). The sensitivity analysis showed that, as GAM and NGAM increased, the growth rate decreased while rates of oxygen uptake and acetate production increased. The impact was much greater in GAM than in NGAM, which is consistent with the previous sensitivity analysis of GAM and NGAM under high glucose uptake condition [48]. Notably, acetate production rate increased gradually when GAM was greater than 52.3 mmol gDCW^-1^ h^-1^ when NGAM was fixed to 5.17 mmol ATP gDCW^-1^ h^-1^, due to the oxygen limitation.

Since metal composition of K-12 was assumed in iHK1487 due to the lack of strain-specific experimental data, we evaluated the influence of each of the metal components on the predicted rates of cell growth, oxygen uptake, and acetate production (S2B Fig). During the sensitivity analysis with the glucose uptake rate of 10 mmol gDCW^-1^ h^-1^, only one metal component was evaluated by varying its proportion in the biomass equation (±20%). All the predicted rates remained almost unchanged depending on any of the metal component, demonstrating negligible impact of the metal composition on the prediction power of the model. The biomass equation having strain-specific parameters (GAM, NGAM, and GC content) is given in S5 Table.

### Phenome analysis

We used PMs to monitor various phenotypic reactions to exogenous compounds and environmental changes [49]. Among the 190 different sources of carbon (PM1 and PM2), BL21(DE3) grew on 91 sources (Fig 1 and S6 Table). The differences in carbon source utilization of BL21(DE3), K-12 MG1655, and B REL606 are summarized in S3 Fig.

**Fig 2:**
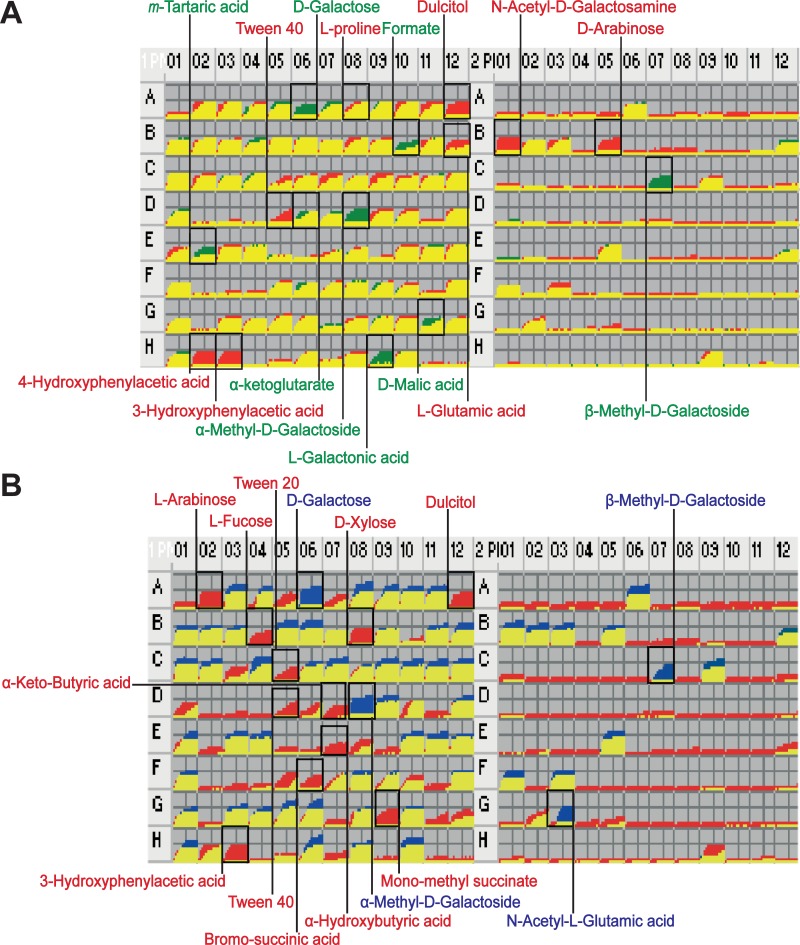
Comparison of carbon source utilization of *E*. *coli* BL21(DE3) and K-12 MG1655. Growth curves in all the cells of the PM1 and PM2 are shown for *E*. *coli* BL21(DE3) (red) and *E*. *coli* K-12 MG1655 (green) (**A**), and for *E*. *coli* BL21(DE3) (red) and *E*. *coli* B REL606 (blue) (**B**).

PM comparisons were made for BL21(DE3) versus K-12 MG1655 (Fig 2A) and BL21(DE3) versus B REL606 (Fig 2B). Compared to K-12, BL21(DE3) grew on 16 carbon sources such as galactitol, 3-HPA, 4-HPA, L-arginine, glycine, L-proline, L-glutamate, N-acetylgalactosamine, and D-arabinose, whereas it did not grow on six carbon sources, namely, *m*-tartaric acid, D-malic acid, L-galactonic acid-γ-lactone, D-galactose, α-methyl-D-galactoside, and β-methyl-D-galactoside. BL21(DE3) and K-12 showed no difference in growth on 13 out of the 17 amino acids used as carbon source (S6 Table). BL21(DE3) grew slowly on α-ketoglutarate, whereas it grew rapidly on L-glutamate, L-proline, L-arginine, and glycine. Remarkably, BL21(DE3) outgrew the B strain of REL606 on 20 carbon sources, including galactitol, L-arabinose, L-fucose, D-xylose, 3-HPA, L-arginine, and glycine, whereas it did not grow on D-galactose, N-acetyl-L-glutamic acid, α-methyl-D-galactoside, and β-methyl-D-galactoside.

We could not assess the usability of nutrients in the PM plates designed for testing the utilization of different sources of nitrogen (PM3), phosphorus, and sulfur (PM4), and auxotrophic supplements (PM5 to PM8). According to the recommendation from the manufacturer (Biolog Inc.), the PM tests for PM3 to PM8 were performed using sodium pyruvate in triplicate and sodium succinate in duplicate as the main carbon source. When sodium pyruvate was used as the carbon source, BL21(DE3) grew in all the cells of PM3 to PM8, including those of the negative control (S4 Fig). However, for sodium succinate, BL21(DE3) manifested growth defects in most of the PM cells. This is unexpected as BL21(DE3) grew on succinate and pyruvate as the carbon source in PM1 and the reason is not immediately obvious. In BL21(DE3), *dcuS* encoding C_4_-dicarboxylate-sensing histidine kinase is frameshifted. BL21(DE3) grew on succinate contained in PM1 slightly slower than K-12. It was reported that *E*. *coli* B REL606 having frameshifted *dcuS* did not grow on succinate of PM1 and showed growth defects in many wells of the PM3 to PM8 plates with succinate as a carbon source [3]. The *dcuSR* mutant of K-12 also showed many growth defects with the PM3 to PM8 plates when using succinate as a carbon source [50]. DcuS is the positive regulator of C_4_-dicarboxylate transporter gene (*dcuB*) whose expression depends on the initial concentration of C_4_-dicarboxylates in the medium [42,51]. Probably, in *dcuS* mutant, the concentration of succinate added to PM3 to PM8 plates became too high for *dcuB* expression. It is more difficult to explain growth in all the PM3 to PM8 wells. Probably, there might be crosstalk regulation between different areas of metabolism [52]. It was reported that the usability of nitrogen sources in PM tests varied with the carbon source used [53]. However, in the case of pyruvate as a carbon source, BL21(DE3) grew even in the negative control wells. Therefore, the reason is hard to be explained without knowing the exact concentration and composition of the culture media dried onto the bottom of each PM well which is confidential and proprietary by the PM manufacturer.

BL21(DE3) was more susceptible than K-12 to various stressful conditions caused by changes in osmolality (PM9) and pH (PM10), and inhibitory compounds such as antibiotics, antimetabolites, and other inhibitors (PM11 to PM20) (S4 Fig). BL21(DE3) did not grow in 40 PM cells containing enoxacin, nalidixic acid, 2,2' dipyridyl, furaltadone, sodium metavanadate, carbenicillin, sodium orthovanadate, protamine sulfate, sulfisoxazole, and ciprofloxacin, whereas it grew in 224 cells containing amikacin, colistin, capreomycin, nafcillin, erythromycin, neomycin, gentamicin, kanamycin, paromomycin, tobramycin, fluoroorotic acid, spectinomysin, ampicillin, 5-fluorouracil, geneticin, cesium chloride, glycine, thallium [I] acetate, moxalactam, sodium arsenate, piperacillin, EGTA, procaine, cefmetazole, D-cycloserine, nordihydroguaiaretic acid, oleandomycin, zinc chloride, phosphomycin, streptomycin, 5-azacytidine, rifamycin, chromium [III] chloride, ferric chloride, L-glutamic acid-γ-hydroxamate, thiosalicylate, hygromycin, ethionamide, 4-aminopyridine, 3-amino-1,2,4-triazole, tannic acid, chlorambucil, phenylarsine oxide, trifluorothymidine, sodium *m*-arsenite, triclosan, myricetin, 3,5-fluoro-5'-deoxyuridine, umbelliferone, disulphiram, phenyl-methyl-sulfonyl fluoride, carbonyl cyanide-*4*-(trifluoromethoxy)phenylhydrazone (FCCP), hydroxylamine, dihydrostreptomycin, apramycin, and crystal violet.

Unlike K-12, BL21(DE3) did not grow in 132 cells containing high concentration of amoxicillin, minocydine, enoxacin, nalidixic acid, chloramphenicol, penicillin G, oxacillin, benzethonium chloride, oxolinic acid, cobalt [II] chloride, trifluoperazine, acriflavine, furaltadone, nitrofurantoin, carbenicillin, domiphen bromide, nitrofurazone, menadione, puromycin, protamine sulfate, diamide, cinoxacin, caffeine, cefoperazone, tinidazole, plumbagin, phenethicillin, captan, orphenadrine, thioridazine, ciprofloxacin, amitriptyline, and ornidazole. BL21(DE3) grew in 28 cells containing colistin, gentamicin, kanamycin, polymyxin B, sodium arsenate, sodium *m*-arsenate, and erythromycin (S4 Fig), whereas K-12 did not. *E*. *coli* B strains have enhanced membrane permeability and have been widely used for mutagenic assays and toxicological studies [54]. Compared to *E*. *coli* K-12, sensitivity to a wider range of antibiotics was also observed for *E*. *coli* B REL606 [3]. This is probably due to differences in the lipopolysaccharide core composition and expression of outer membrane proteins, resulting in alterations in screening barriers that control import and export of antibiotics. Similar to other B strains, BL21(DE3) is deficient in the Lon protease, which is associated with bacterial evolution to antibiotic resistance [55] and has additional type II secretion system [24]. As current understanding of the mechanism underlying the antibiotic sensitivity of BL21(DE3) is far from complete, experimental data can be used to refine and extend the model.

### Validation and revision of the metabolic model

The reconstructed model was validated by comparing the simulated growth from FBA using the model with experimental growth from PM tests (S7 Table). The simulated and experimental growths were not matched for 34 carbon sources. Among them, four false negatives (growth from PM tests and non-growth from simulation) were reflected to modify the model (Table 2). We added three transport reactions and one exchange reaction of fucose and glyoxylate, 12 metabolic reactions, two transport reactions, and two exchange reactions for 3-HPA metabolism, and one metabolic reaction for melibiose utilization. Two reactions for *myo*-inositol metabolism encoded by putative genes (*yhiJ* and *yidK*) were removed as BL21(DE3) did not grow on *myo*-inositol in the PM tests. In conclusion, the simulated growth qualitatively agreed with PM results for 160 out of 190 carbon sources, and the predictive accuracy of our model can be considered to be 84.2%, representing 4.7% increase over 79.5% accuracy of the iECD_1391.

**Table 2:** Different phenotypes of *E*. *coli* BL21(DE3) and K-12 MG1655, and modifications of the corresponding reactions.

Carbon sources	PM^a^ BL21(DE3)/K-12	Prediction^b^ BL21(DE3)/K-12	Associated reactions^c^	Modification
D-Galactose	-/+	-/+	UDPG4E, GALM2pp, and UGLT	Deletion
β-Phenylethylamine	-/-	-/+	PACCOAE, DHACOAH, OXDHCOAT, REPHACCOAI, HADPCOADH3, 3OXCOAT, PACCOAL, OXCOAHDH, PACOAT, HPACOAT, and 2HPTCOAT	Deletion
4-Hydroxyphenylacetic acid	+/-	+/-	4H3M, 34DH23OR, 5C2HMSO, 5C2HMDI, 5O3E125TC, HPAG, 2OH3E17DH, HPAH, HPAI, HPAP, HPAtex, and EX_4hphac_e	Addition
3-Hydroxyphenylacetic acid	+/-	+/-	3H3M, 3HPAP, 3HPAtex, and EX_3hphac_e	Addition
N-Acetylgalactosamine	+/-	+/-	ACGALptspp, and ACGAL6PI	Addition
D-Arabinose	+/-	+/-	RBK_D1, DARBabcpp, DARBt2rpp, DARBt3ipp, DARBtex, and EX_arab-D_e	Addition
Glyoxylic acid	+/+	+/-	GLXtmp, GLXt2_3pp, and EX_glx_e	Addition
L-Fucose	+/+	+/+	FUCtpp	Addition
D-Malic acid	-/+	-/+	MALDt2_2pp	Deactivated
L-Galactonic acid-γ-lactone	-/+	-/+	GALCTLO, GALCTNLtex, and EX_galctn__L_e	Deactivated

The complete list of experimental and simulated cell growth on carbon sources is available in S7 Table.

^a^Cell growth from PM tests are categorized into non-growth (‘-‘) or growth (‘+’) based on statistical analysis of five growth replicates of each strain (see S7 Table for details).

^b^Simulated cell growth on each carbon source by flux balance analysis: non-growth (‘-’), growth (‘+’).

^c^Detailed information for the reactions is listed in S1 Table.

^13^C metabolic flux analysis (^13^C-MFA) is the robust approach to experimentally quantify the metabolic fluxes in central carbon metabolism [56]. We used published ^13^C-MFA data to evaluate prediction accuracy of a metabolic network model. As shown in S5A Fig, the predicted fluxes using iHK1487 were well fitted to ^13^C fluxes of BL21 [57] (*r*^2^ = 0.95). Since BL21(DE3) and K-12 have the same metabolic gene repertoire in central carbon metabolism with the exception of absence of *pgl* in BL21(DE3), K-12 without PGL reaction can have flux distribution in central metabolism similar to BL21(DE3). Remarkably, the predicted fluxes using iHK1487 also agreed well with ^13^C fluxes of the deletion mutant of *pgl* in K-12 [58] (*r*^2^ = 0.95) (S5B Fig), implying that absence of *pgl* is an important intrinsic property of BL21(DE3) in the central carbon metabolism. The excellent agreement between *in vivo* measurements and *in silico* predictions indicate iHK1487 yielded fairly accurate flux predictions.

### Analysis of co-factor balance in energy metabolism

**Fig 3:**
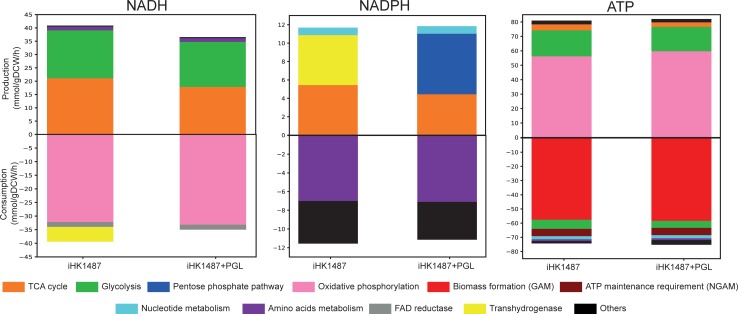
Quantitative analysis of co-factor balances (NADH, NADPH, and ATP) in FBA using iHK1487. For each of the co-factors, the production and consumption rates predicted using iHK1487 are compared with those using iHK1487 added with the PGL reaction (iHK1487 + PGL). The maximum uptake rates of glucose and oxygen were set to 10 mmol gDCW^-1^ h^-1^ and 18.5 mmol gDCW^-1^ h^-1^, respectively. Cellular processes associated with production and consumption of co-factors are color-coded. “Others” denotes sum of the cellular processes with minor contribution.

To expand insights on energy metabolism, we calculated production and consumption rates of major co-factors of the redox carriers (NADH and NADPH) and the energy carrier (ATP). As the major feature of BL21 in the central metabolism is the absence of *pgl* involved in the oxidative PPP, the simulation results from iHK1487 (representing wild-type BL21(DE3)) were compared with those from iHK1487 added with the PGL reaction (iHK1487 + PGL) (representing BL21(DE3) having the active PGL reaction) (Fig 3). For FBA, the maximum uptake rates of glucose and oxygen were set to 10 mmol gDCW^-1^ h^-1^ and 18.5 mmol gDCW^-1^ h^-1^, respectively. Under the simulation condition, the optimal growth rate was similar in both cases (0.82 h^-1^ for iHK1487 and 0.831 h^-1^ for iHK1487 + PGL). For the aerobic growth on glucose, total production rates of NADPH and ATP were similar in both cases. However, total production rate of NADH were 12% higher in iHK1487 (40.5 mmol gDCW^-1^ h^-1^) than in iHK1487 + PGL (36.2 mmol gDCW^-1^ h^-1^), due to the higher flux through glycolysis and TCA cycle in iHK1487. And then, the increased NADH in iHK1487 was converted to NADPH by transhydrogenase to compensate the loss of NADPH production by the block of the oxidative PPP. Total ATP production rates were very similar in iHK1487 (78.3 mmol gDCW^-1^ h^-1^) and iHK1487 + PGL (79.4 mmol gDCW^-1^ h^-1^), and the majority of ATP was produced from oxidative phosphorylation (> 71%) via oxidation of NADH. Taken together, these results demonstrate that absence of PGL reaction in BL21(DE3) considerably affects the way of NADH/NADPH generation.

### Gene essentiality analysis

FBA was performed to predict the reactions essential for cell growth of BL21(DE3) and K-12 strains. In BL21(DE3) and K-12, 195 genes were predicted to be essential. In addition, BL21(DE3) harbored four more essential genes (*argI*, *cysU*, *cysW*, and *cysA*) which were not predicted to be essential in K-12. Ornithine carbamoyltransferase is involved in the L-arginine biosynthetic pathway, which is encoded by two genes in K-12 (*argF*, and *argI*) and by one gene in BL21(DE) (*argI*) [24,59]. In the K-12 metabolic model (iML1515) [25], sulfate is annotated to be transported by two operons of *cysPUWA*, which encode an ATP-dependent sulfate/thiosulfate uptake system, and by *modABC*, which encodes a molybdate ABC transporter. The *modABC* operon is missing in BL21(DE3) [24], and hence *cysUWA* becomes essential for BL21(DE3). All the 199 genes associated with 382 reactions predicted to be essential for BL21(DE3) were expressed in more than one condition among the five culture/growth conditions of our transcriptome analysis [24] (S8 Table).

Among 69 metabolic genes involved in central metabolic pathways, nine were reported to be essential for K-12 growing aerobically on glucose [25]. Of the nine essential genes, two genes involved in TCA cycle (*gltA* encoding citrate synthase and *icd* encoding isocitrate dehydrogenase) were predicted to be essential from FBA using iML1515 of K-12 [25], which was the same when using iHK1487 (S8 Table). The consistent results might imply that lack of PGL reaction is not critical to overall energy metabolism and can be easily reconciled by rerouting fluxes in the metabolic network. The remaining seven genes (*lpd*, *gapA*, *eno*, *fbaA*, *pgk*, *pfkA*, and *tpiA*) represent false positives (i.e. predicted growth while experimental non-growth). The false growth prediction can be used to refine the model when more biochemical information is available [4].

### Use of transcriptome data for metabolic simulation

Integrating transcriptome data into constraint-based models of metabolic network can improve flux prediction [60–62]. In this study, we deactivated some reactions by constraining their flux to zero in FBA simulation if their associated genes were not expressed from the strand-specific transcriptome profiles of BL21(DE3) for the five different culture conditions (exponential and stationary phases, and complex and defined media) [24]. In the transcriptome study, total RNA was directly labeled and hybridized with oligonucleotide probes on the single color genome-wide tiling array, and thus, the probe intensities were proportional to the gene expression intensities.

**Fig 4:**
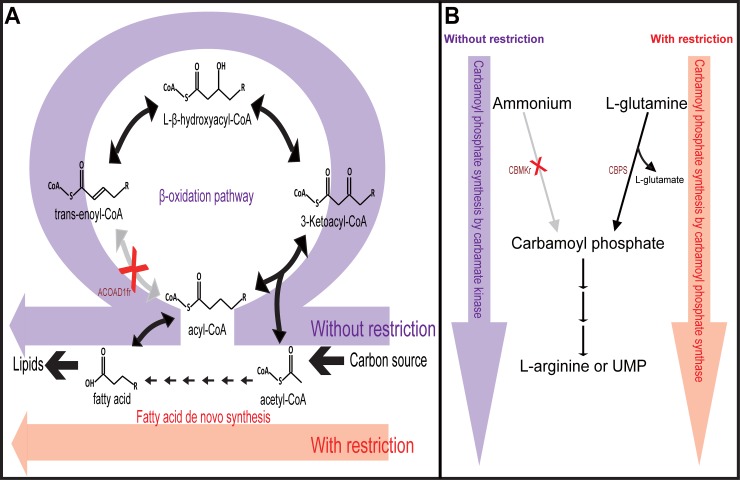
Comparison of the predicted flux distributions using FBA with and without additional constraints from transcriptome data. Shown are fluxes through the biosynthetic pathways for lipid synthesis (**A**) and carbamoyl phosphate synthesis (**B**). The condition-specific constraints on fluxes were inferred from the previous transcriptome data at the exponential phase of BL21(DE3) grown in a defined medium [24]. The flux distribution from the unrestricted FBA is colored purple and that from the restricted FBA is colored red.

When all the 238 fluxes corresponding to the 336 genes that were not expressed at the exponential phase in the defined medium were set to zero, the growth objective value was almost identical to that when such restriction was not imposed on the fluxes. Regarding flux distribution, most of the flux values did not change (< 5%), 44 fluxes disappeared or decreased, and 57 appeared *de novo* or were increased (S9 Table). Notably, 34 fluxes involved in the β-oxidation pathway for the fatty acid degradation disappeared or decreased, and instead, 43 fluxes related to *de novo* biosynthetic pathways of fatty acid newly appeared or increased (Fig 4A). Another example is that carbamoyl phosphate was synthesized from L-glutamine, not from ammonium (Fig 4B). Previous *E*. *coli* proteome analysis showed that enzymes of the β-oxidation cycle (FadA, and FadB) were not synthesized in flask cultures using defined medium without fatty acid [63], which agrees with the flux distribution from the restricted FBA. The modification that contributed to the improvement observed in fatty acid biosynthesis was the deactivation of the acyl-CoA dehydrogenase (ACOAD1fr) encoded by the putative acyl-CoA dehydrogenase (*ydiO*) in the β-oxidation cycle. This case study demonstrates that the prediction power of the metabolic model can be improved by incorporating transcriptome data into metabolic simulation.

## Discussion

**Fig 5:**
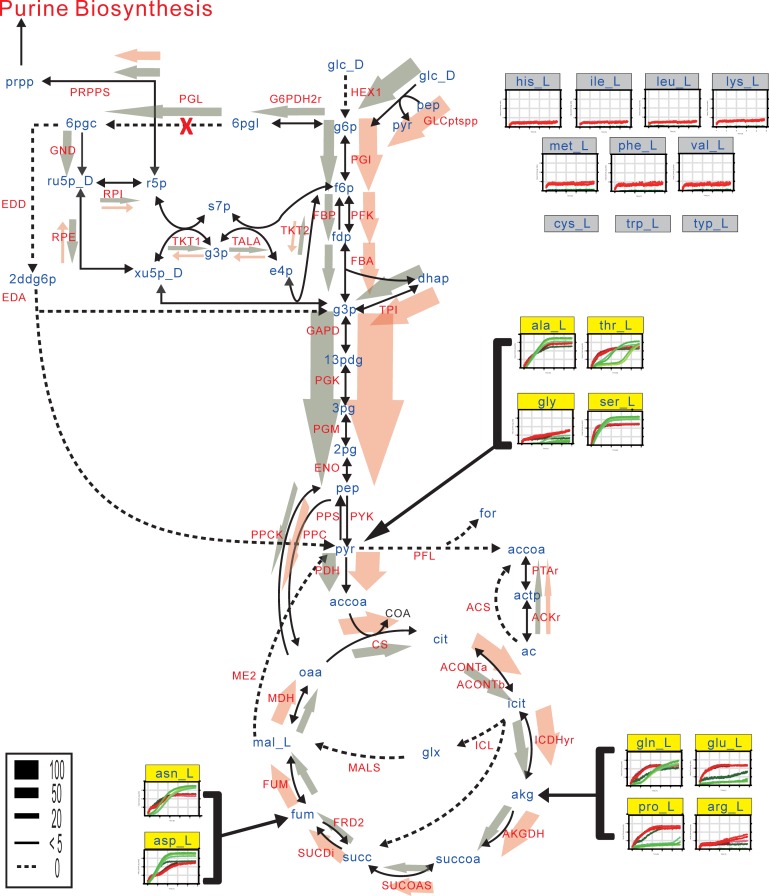
Distribution of the predicted fluxes in central metabolic pathways of *E*. *coli* BL21(DE3) and K-12 MG1655. Each arrow denotes the direction of the reaction in proportion to the associated flux value of BL21(DE3) (orange) and K-12 MG1655 (dark green). Individual thumbnail graphs represent growth of the BL21(DE3) (red) and K-12 MG1655 (green) cells on particular amino acid as the carbon source, demonstrated by phenotype microarray tests. The x-axes denote culture time (up to 48 hours) and the y-axes represent cell growth in arbitrary unit. The header background of each thumbnail graph is yellow if the corresponding amino acid can be used as a carbon source in iHK1487, and is grey otherwise. Abbreviations are shown in S5 Table.

The major genetic difference in the central metabolism between BL21 and K-12 strains is the lack of *pgl*, which encodes 6-phosphogluconolactonase (Pgl) that is involved in the oxidative branch of the pentose phosphate pathway (PPP) [27,64]. Absence of PGL in BL21(DE3) can limit supply of NADPH for reducing power requirements, as well as those of ribose-5-phosphate and erythrose-4-phosphate for nucleotide and amino acid biosynthesis [64–66]. Simulation of the BL21(DE3) metabolic model with glucose as the sole carbon source showed that the carbon flux did not flow through the oxidative branch of the PPP, but channeled into the glycolytic pathway (Fig 5). This enhanced the flux through the glycolytic pathway and TCA cycle. Ribulose-5-phosphate in the PPP was produced via the transaldolase reaction catalyzed by TalA and TalB, rerouting the PPP, which agreed with the previous simulation of the BL21 metabolic model [27]. Our simulation showed that the rerouted flux through the transaldolase reaction could generate ribose-5-phosphate and erythrose-4-phosphate, leading to undiminished flux through the nucleotide biosynthetic pathway.

*E*. *coli* BL21(DE3) is widely used for the mass-production of recombinant proteins [21]. Comparative multi-omics analysis of *E*. *coli* B and K-12 strains showed that most of the genes involved in amino acid biosynthetic pathways were expressed more in B strains [3]. In addition, phenome data and simulation results demonstrated that BL21(DE3) and K-12 differed in the pattern of amino acid utilization. Among the 17 kinds of amino acids tested in the PM plates, BL21(DE3) and K-12 used 10 and six amino acids as carbon sources, respectively. The extracellular amino acids can be metabolized to generate three precursor intermediates in the central catabolic pathways: from alanine, threonine, glycine, and serine to pyruvate; from asparagine and aspartate to fumarate; from glutamine, glutamate, proline, and arginine to α-ketoglutarate (Fig 5). However, K-12 did not grow on glutamate, proline, and arginine. The differences in amino acid utilization patterns between BL21(DE3) and K-12 do not appear to be the result of the different amino acid sequences of the amino acid transporters, as the protein sequences of the transporters of glutamate, proline, and arginine are nearly identical in BL21(DE3) and K-12. Glutamate and glutamine are the key intermediates in cellular nitrogen metabolism [67]; however, previous reports showed that wild-type *E*. *coli* could not grow on glutamate [68] and grew poorly on glutamine as the carbon source [69]. The efficient utilization of glutamate and glutamine as carbon sources demonstrates the enhanced capacity of BL21(DE3) for nitrogen metabolism than other *E*. *coli* strains.

Interestingly, compared to K-12, BL21(DE3) did not grow well on α-ketoglutarate in PM tests (Fig 2A and S6 Fig). The protein sequences of the α-ketoglutarate/H^+^ symporter (KgtP) of BL21(DE3) and K-12 MG1655 are identical. α-Ketoglutarate lies at the intersection between the carbon and nitrogen metabolic pathways and is a master regulator metabolite for nitrogen-assimilatory reactions [70]. In addition, unlike K-12, BL21(DE3) does not contain *gltF* adjacent to the *gltBD* operon encoding glutamate synthase, which converts α-ketoglutarate to glutamate [24]. GltF was suggested to play a regulatory role in nitrogen catabolism and ammonium transport [71]. Simulation of amino acid utilization predicted that both BL21(DE3) and K-12 can grow on glutamate, proline, and arginine, which only were in agreement with the PM results of BL21(DE3). These observations imply that the regulation between carbon and nitrogen metabolism in BL21(DE3) differs from that of K-12 MG1655.

The automatically reconstructed metabolic model should be considered as a draft model, which requires extensive expert review. iECD_1391, the metabolic model of BL21, which is the predecessor of BL21(DE3), was first reconstructed in an automatic manner [26]. The BL21 model was updated [27]; however, it was similar to the previous version except for removal of reactions falsely associated with pseudogenes or ambiguous genes. We observed that the current iECD_1391 model still contains many metabolic reactions associated with pseudogenes or genes with incomplete annotation (S10 Table). However, our model, iHK1487, incorporated the recent genome annotation of BL21(DE3) [24] and the recent version of the metabolic model of the closely related strain, K-12 MG1655. The iHK1487 was highly manually curated and was revised using phenome data. This effort increased the gene coverage and accuracy of the metabolic model (Table 1).

Gene expression data can provide experimental evidences for the addition of model entities. We also used the transcriptome data to improve the flux prediction in a condition-specific manner (Fig 4). Set of genes that were not expressed in the defined medium were identified from the transcriptome profiles [24], and their corresponding fluxes were constrained to zero. The additional constraints can reduce the size of the solution space of the flux distribution, which is helpful in predicting the precise flux state in a cell.

We showed that addition of BL21(DE3)-specific regulation into the metabolic model made the metabolic model congruent with the carbon source utilization data obtained from PM tests. As the regulatory rules of the current model were limited to two regulatory genes (*IgoR* and *dcuS*) affecting 11 transport reactions, the iHK1487 can be further improved by integrating more regulatory rules. For example, FBA using iHK1487 predicted no flux through the glyoxylate shunt pathway regardless of glucose concentration (Fig 5 and S5 Fig). This is contrasting to the previous experimental observation that the glyoxylate shunt in BL21 was highly active regardless of glucose concentration, resulting in low acetate excretion even under high glucose concentration, and fast growth thereby [72]. Compared to K-12, BL21 has no unique metabolism to use glyoxylate shunt for the glucose metabolism. However, BL21 has been suggested to have different regulations affecting the glyoxylate shunt activity, such as constitutive expression of *cra* (encoding catabolic repressor/activator) [73], reduced expression of *arcA* (global regulator), *iclR* (repressor of *aceBAK* operon) [74], and induction of small RNA degrading mRNA ptsG under high glucose concentration [75], although the exact regulatory mechanism is still not clear. It should be mentioned that the discrepancy between FBA and real fluxome can be due to sub-optimal cell metabolism (i.e., few metabolic pathways operate at full efficiency), especially when complex regulatory mechanisms are involved. Therefore, incorporation of the exact regulatory rules into the iHK1487 will increase the prediction accuracy for the glucose metabolism.

BL21(DE3) is one of the primary choices for overproduction of recombinant proteins, biofuels, and biorefineries [21]. The genome-wide metabolic network model is useful for understanding the genotype-phenotype relationships and designing and optimizing microbial hosts through modulation of the metabolic flux [1,2,4,76]. Until now, development of bioprocesses using BL21(DE3) based on modeling and simulation has been limited because of the lack of comprehensive metabolic network models. The iHK1487 will be pivotal for designing host strains with customized genomes and developing rational fermentation strategies. As our knowledge on BL21(DE3) is far from complete and the omics-based systems approach of the strain is in an early stage [3,21,24], the iHK1487 will serve as a framework for integration of various omics data and metabolic engineering of BL21(DE3).

## Supporting information

S1 Fig Determination of GAM and NGAM in iHK1487 using chemostat data of *E*. *coli* B/r. (DOCX)

S2 Fig Influences of energy maintenance requirements (GAM and NGAM) and metal cofactors (MoO4-2, Cu^+2^, Mn^+2^, Zn^+2^, Ni^+2^, and Co^+2^) on the predicted rates of cell growth, oxygen uptake, and acetate production. (DOCX)

S3 Fig Comparison of carbon source utilization of BL21(DE3), K-12 MG1655, and B REL606. (DOCX)

S4 Fig Comparison of phenotype microarrays (PMs) of *E*. *coli* BL21(DE3) and K-12 MG1655. (DOCX)

S5 Fig Flux distribution in central carbon metabolism by *in vivo* measurements and *in silico* predictions. (DOCX)

S6 Fig Comparison of carbon source utilization of *E*. *coli* BL21(DE3) and K-12 MG1655 tested in phenotype microarray (PM) plates 1 and 2. (DOCX)

S1 Table Metabolic reactions modified in the BL21(DE3) model (iHK1487) compared to the K-12 model (iML1515). (XLSX)

S2 Table Metabolites present only in the BL21(DE3) model (iHK1487) compared to the K-12 model (iML1515). (XLSX)

S3 Table Expression of genes associated with metabolic reactions present only in the BL21(DE3) model (iHK1487) compared to the K-12 model (iML1515). (XLSX)

S4 Table K-12 metabolic reactions that are not included in the BL21(DE3) model (iHK1487). (XLSX)

S5 Table iHK1487 in EXCEL format. (XLSX)

S6 Table Phenotypic comparison of BL21(DE3), K-12 MG1655, and B REL606 for carbon source utilization. (XLSX)

S7 Table Phenotypic comparison of BL21(DE3) and K-12 MG1655 and *in silico* predictions of cell growth on carbon sources. (XLSX)

S8 Table Predicted essential genes of *E*. *coli* BL21(DE3). (XLSX)

S9 Table Comparison of flux distributions simulated with and without condition-specific constraints on fluxes. (XLSX)

S10 Table Metabolic reactions of iECD_1391 that are not included in iHK1487. (XLSX)

S1 File iHK1487 in SBML format. (XML)
