# Supplementary material for: Metabolic network reconstruction and phenome analysis of the industrial microbe, Escherichia coli BL21(DE3)
Source: PLoS One. 2018 Sep 21;13(9):e0204375. doi: 10.1371/journal.pone.0204375 (PMC6150544; doi:10.1371/journal.pone.0204375)
Supplement: S1 Fig — (DOCX) [file pone.0204375.s001.docx]

**S1 Fig. Determination of GAM and NGAM in iHK1487 using chemostat data of *E. coli* B/r.** The experimental plot of growth rate versus glucose uptake rate was generated using parameters of maintenance coefficient and maximum ATP yield which were determined from glucose-limited chemostat cultures of *E. coli* B/r growing aerobically on minimal medium (Reiling et al., J Biotechnol, 2:191-206, 1985). For FBA, the maximum oxygen uptake rate was set to 18.5 mmol gDCW^-1^ h^-1^.

**
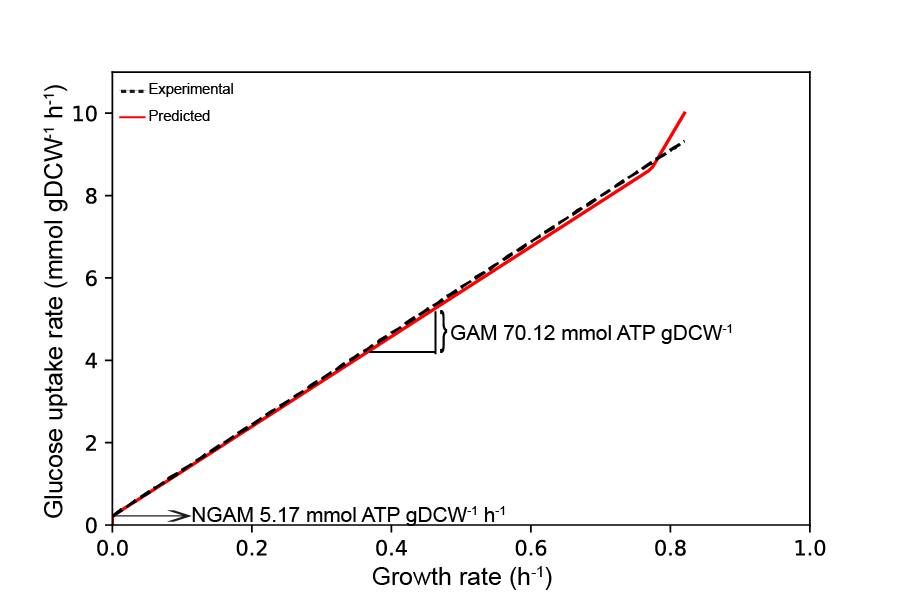
**
