# Supplementary material for: Metabolic network reconstruction and phenome analysis of the industrial microbe, Escherichia coli BL21(DE3)
Source: PLoS One. 2018 Sep 21;13(9):e0204375. doi: 10.1371/journal.pone.0204375 (PMC6150544; doi:10.1371/journal.pone.0204375)
Supplement: S2 Fig — (DOCX) [file pone.0204375.s002.docx]

**S2 Fig. Influences of energy maintenance requirements (GAM and NGAM) and metal cofactors (MoO_4_^-2^, Cu^+2^, Mn^+2^, Zn^+2^, Ni^+2^, and Co^+2^) on the predicted rates of cell growth, oxygen uptake, and acetate production.** The maximum glucose uptake rate was set to 10 mmol gDCW^-1^ h^-1^ for FBA. GAM and NGAM from various sources are summarized in the table. Predicted rates are plotted by varying GAM and NGAM (**A**) and by varying each of metal cofactors (**B**).

| **ID** | **GAM**  **(mmol ATP gDCW^-1^)** | **NGAM**  **(mmol ATP gDCW^-1^ h^-1^)** | **Source** |
| --- | --- | --- | --- |
| a | 59.81 | 8.39 | iAF1260 of K-12  (Feist et al., Mol Syst Biol, 3:121, 2007) |
| b | 53.95 | 3.15 | iJO1366 of K-12  (Orth et al., Mol Syst Biol, 7:535, 2011) |
| c | 75.55 | 6.86 | iML1515 of K-12  (Monk et al., Nat Biotechnol, 35:904-908, 2017) |
| d | 70.12 | 5.17 | iHK1487 of BL21(DE3) (in this study) |

**
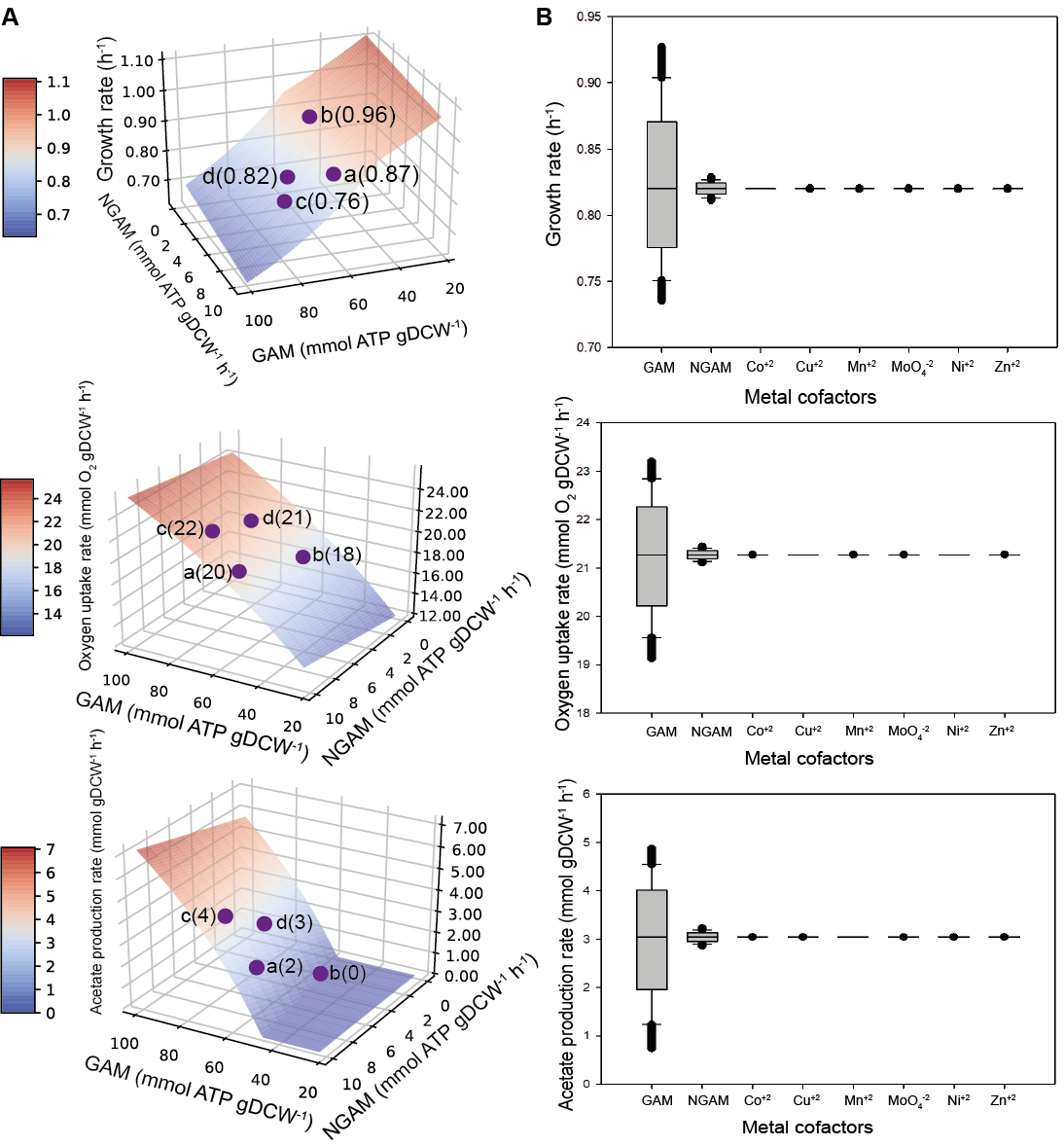
**
