# Supplementary material for: Metabolic network reconstruction and phenome analysis of the industrial microbe, Escherichia coli BL21(DE3)
Source: PLoS One. 2018 Sep 21;13(9):e0204375. doi: 10.1371/journal.pone.0204375 (PMC6150544; doi:10.1371/journal.pone.0204375)
Supplement: S3 Fig — (DOCX) [file pone.0204375.s003.docx]

**S3 Fig. Comparison of carbon source utilization of BL21(DE3), K-12 MG1655, and B REL606.** The substrates are (**A**) 'acetic acid', 'maltotriose', 'mucic acid', 'fumaric acid', 'D-mannose', 'glycolic acid', 'D-gluconic acid', 'D-galactonic acid-γ-lactone', 'inosine', 'ala-gly', 'D-galacturonic acid', 'propionic acid', 'D-mannitol', 'D-fructose', 'thymidine', 'N-acetyl-β-D-mannosamine', '2′-deoxy-adenosine', 'α-D-lactose', 'uridine', 'α-D-glucose-1-phosphate', 'L-lactic acid', 'L-glutamine', 'glyoxylic acid', 'D,L-malic acid', 'succinic acid', 'D-glucose', 'gly-asp', 'D,L-α-glycerol-phosphate', 'L-rhamnose', '3-O-β-D-galactopyranosyl-D-arabinose', 'D-glucuronic acid', 'D-saccharic acid', 'gly-glu', 'N-acetyl-D-glucosamine', 'D-fructose-6-phosphate', 'D-lactic acid methyl ester', 'β-D-allose', 'glycerol', 'methyl pyruvate', 'D-melibiose', 'D-glucosamine', 'melibionic acid', 'L-malic acid', 'L-threonine', 'gly-pro', 'L-serine', 'D-alanine', 'L-aspartic acid', 'L-alanine', 'pyruvic acid', 'N-acetyl-neuraminic acid', 'L-asparagine', 'D-glucose-6-phosphate', 'adenosine', 'L-lyxose', 'β-methyl-D-glucuronic acid', 'dihydroxyacetone', 'D-sorbitol', 'dextrin', 'D-ribose', 'D-maltose', 'L-alaninamide', 'D-serine', 'Lactulose', 'D-trehalose', and '2,3-butanedione'; (**B**) 'dulcitol', 'tween 20', 'tween 40', 'mono-methyl succinate', '3-HPA', 'glucuronamide', 'β-cyclodextrin', 'arbutin', 'L-arginine', 'glycine', and 'L-ornithine'; (**C**) 'L-arabinose', 'L-fucose', 'D-xylose', 'α-keto-glutaric acid', 'α-keto-butyric acid', 'α-hydroxy-butyric acid', 'β-methyl-D-glucoside', 'Bromo-succinic acid', and '5-keto-D-gluconic acid'; (**D**) 'L-proline', 'L-glutamic acid', '4-HPA', 'N-acetylgalactosamine', and 'D-arabinose'; (**E**) '*m*-tartaric acid', 'D-malic acid', and 'L-galactonic acid-γ-lactone'; (**F**) 'D-galactose', 'α-methyl-D-galactoside', and 'β-methyl-D-galactoside'; (**G**) 'N-acetyl-L-glutamic acid'; (**H**) 'sodium formate', 'D-aspartic acid', 'D-glucosaminic acid', '1,2-propanediol', 'sucrose', 'tween 80', 'α-hydroxy-glutaric acid-γ-lactone', 'adonitol', 'citric acid', '*myo*-inositol', 'D-threonine', 'D-cellobiose', 'tricarballylic acid', 'acetoacetic acid', 'tyramine', 'D-psicose', 'β-phenylethylamine', 'ethanolamine', 'chondroitin sulfate C', 'α-cyclodextrin', 'γ-cyclodextrin', 'gelatin', 'glycogen', 'inulin', 'laminarin', 'mannan', 'pectin', 'amygdalin', 'D-arabitol', 'L-arabitol', '2-deoxy-D-ribose', '*m*-erythritol', 'D-fucose', 'β-gentiobiose', 'L-glucose', 'D-lactitol', 'D-melezitose', 'maltitol', 'α-methyl-D-glucoside', '3-O-methyl-D-glucose', 'α-methyl-D-mannoside', 'β-methyl-D-xylopyranoside', 'palatinose', 'D-raffinose', 'D-salicin', 'sedoheptulosan', 'L-sorbose', 'stachyose', 'D-tagatose', 'turanose', 'xylitol', 'N-acetyl-D-glucosaminitol', 'γ-amino-n-butyric acid', 'δ-amino-valeric acid', 'butyric acid', 'capric acid', 'caproic acid', 'citraconic acid', 'D-citramalic acid', '2-hydroxy-benzoic acid', '4-hydroxy-benzoic acid', 'β-hydroxy-butyric acid', 'γ-hydroxy-butyric acid', 'α-keto-valeric acid', 'itaconic acid', 'malonic acid', 'oxalic acid', 'oxalomalic acid', 'quinic acid', 'D-ribono-1,4-lactone', 'sebacic acid', 'sorbic acid', 'succinamic acid', 'D-tartaric acid', 'L-tartaric acid', 'acetamide', 'L-histidine', 'L-homoserine', 'L-hydroxyproline', 'L-isoleucine', 'L-leucine', 'L-lysine', 'L-methionine', 'L-phenylalanine', 'L-pyroglutamic acid', 'L-valine', 'D,L-carnitine', 'butylamine', 'D,L-octopamine', 'putrescine', '2,3-butanediol', and '3-hydroxy-2-butanone'

**
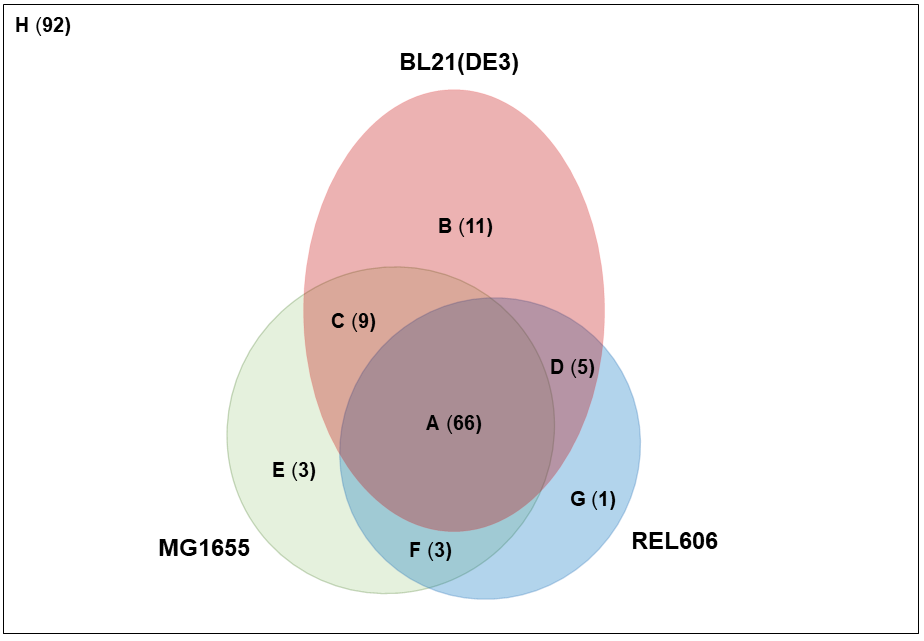
**
