# Supplementary material for: Metabolic network reconstruction and phenome analysis of the industrial microbe, Escherichia coli BL21(DE3)
Source: PLoS One. 2018 Sep 21;13(9):e0204375. doi: 10.1371/journal.pone.0204375 (PMC6150544; doi:10.1371/journal.pone.0204375)

**S4 Fig. Comparison of phenotype microarrays (PMs) of *E. coli* BL21(DE3) and K-12 MG1655.** Growth curves during 48 hours are colored yellow for similar growth of BL21(DE3) and K-12, green for faster growth of K-12, and red for faster growth of BL21(DE3). The PM plates (Biolog Inc.) consist of twenty 96-well microplates containing different sources of carbon (PM1 and PM2), nitrogen (PM3), phosphorus, and sulfur (PM4), auxotrophic supplement (PM5 to PM8), salt (PM9), pH stress (PM10), and inhibitory compounds such as antibiotics, antimetabolites, and other inhibitors (PM11 to PM20). Sodium pyruvate was supplemented as the main carbon source (PM3 to PM8). Detailed information on PM tests can be found at [www.biolog.com](http://www.biolog.com).


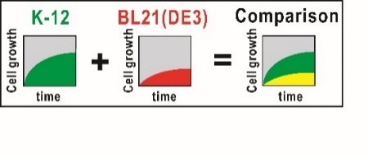

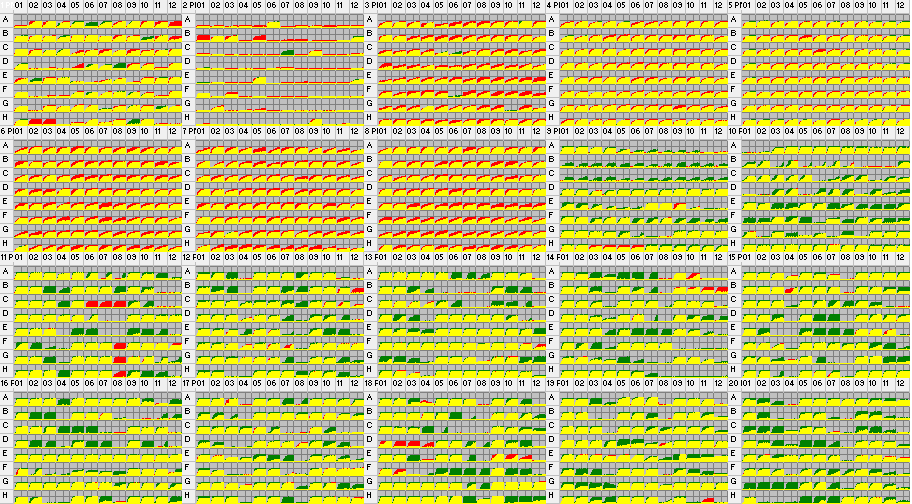

Supplement: S4 Fig — (DOCX) [file pone.0204375.s004.docx]
