# Supplementary material for: Metabolic network reconstruction and phenome analysis of the industrial microbe, Escherichia coli BL21(DE3)
Source: PLoS One. 2018 Sep 21;13(9):e0204375. doi: 10.1371/journal.pone.0204375 (PMC6150544; doi:10.1371/journal.pone.0204375)

**S5 Fig. Flux distribution in central carbon metabolism by *in vivo* measurements and *in silico* predictions.** The predicted fluxes were compared with the calculated fluxes from ^13^C-MFA studies on BL21 (Long et al., Metab Eng, 44:100-107, 2017) (**A**) and on *pgl* mutant of K-12 (Ishii et al., Science, 316:593-597, 2007) (**B**). It should be mentioned that both of ^13^C-MFA studies assumed spontaneous reaction for the missing PGL reaction (Kupor and Fraenkel, J Biol Chem, 247:1904-1910, 1972) in ^13^C calculation. Experimentally determined uptake rates of glucose and oxygen (Long et al., Metab Eng, 44:100-107, 2017; Millard et al., PLoS Comput Biol, 13:e1005396, 2017) (denoted above in each figure) were used for FBA. All fluxes are normalized to 100 units of glucose uptake. On each reaction link, the upper number denotes ^13^C-flux and the lower number represents the predicted flux. Plot of the ^13^C-flux versus predicted flux and the coefficient of determination (*r*^2^) are shown at the bottom. Abbreviations are shown in S5 Table.


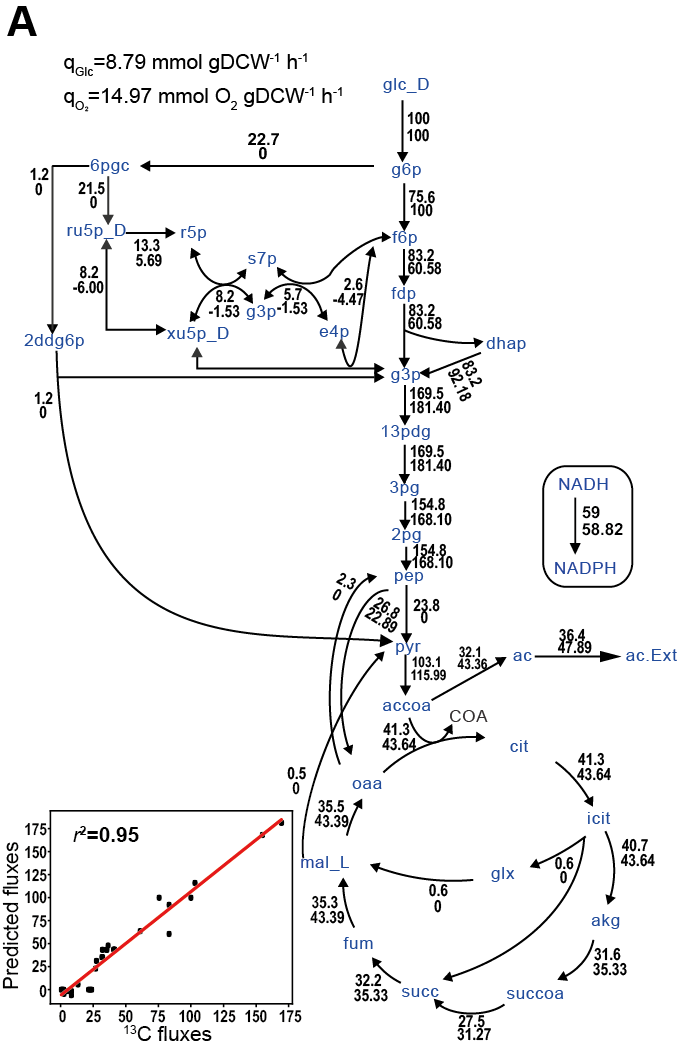


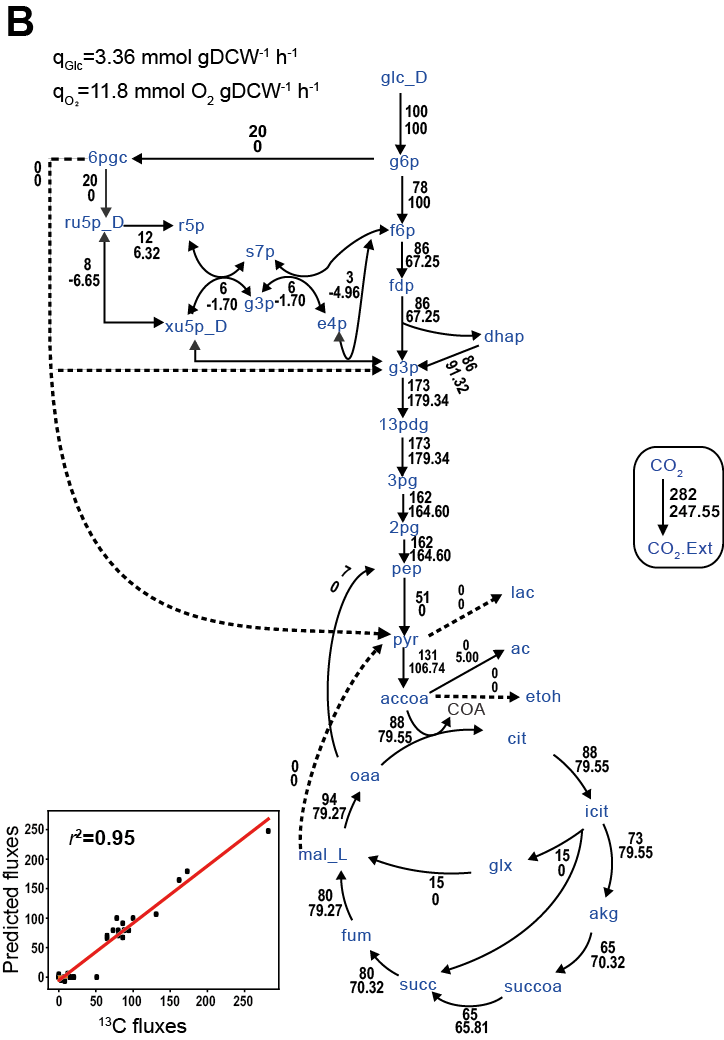

Supplement: S5 Fig — (DOCX) [file pone.0204375.s005.docx]
