# Supplementary material for: Metabolic network reconstruction and phenome analysis of the industrial microbe, Escherichia coli BL21(DE3)
Source: PLoS One. 2018 Sep 21;13(9):e0204375. doi: 10.1371/journal.pone.0204375 (PMC6150544; doi:10.1371/journal.pone.0204375)

**S6 Fig. Comparison of carbon source utilization of *E. coli* BL21(DE3) and K-12 MG1655 tested in phenotype microarray (PM) plates 1 and 2.** The heatmaps were created based on the area under curve (AUC) values of cell growth curves. Five PM replicates of BL21(DE3) (rep1~5) and one replicate of K-12 MG1655 (rep5) were produced from this study. Four PM replicates of K-12 MG1655 (rep1~4) were obtained from the previous study (Yoon et al., Genome Biol, 13:R37, 2012). Individual thumbnail graphs represent cell growth of the BL21(DE3) (red) and K-12 MG1655 (green) on particular amino acids as the carbon source, as shown by PM tests. The x-axes denote culture time (up to 48 hours) and the y-axes represent cell growth in arbitrary units.


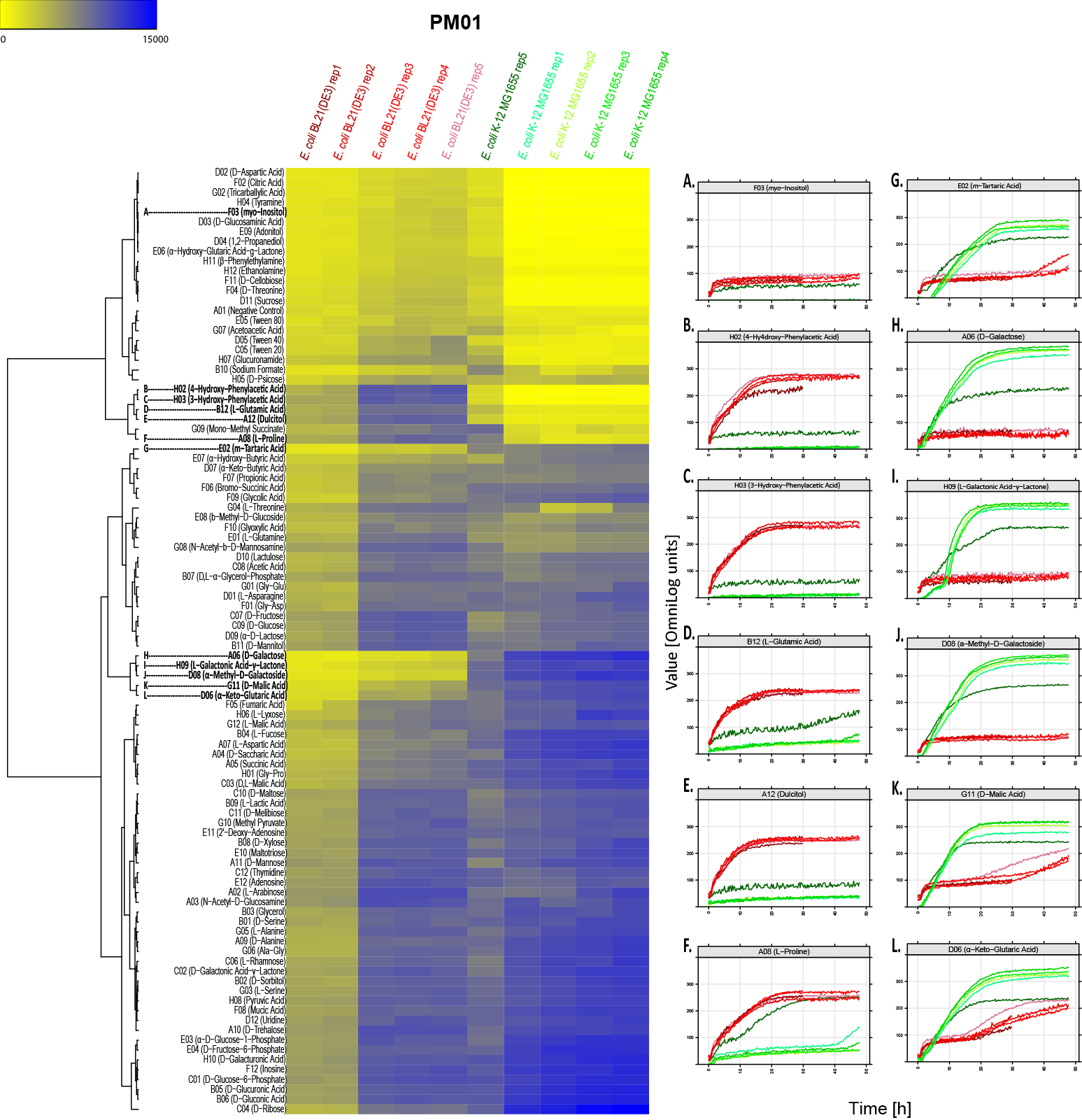


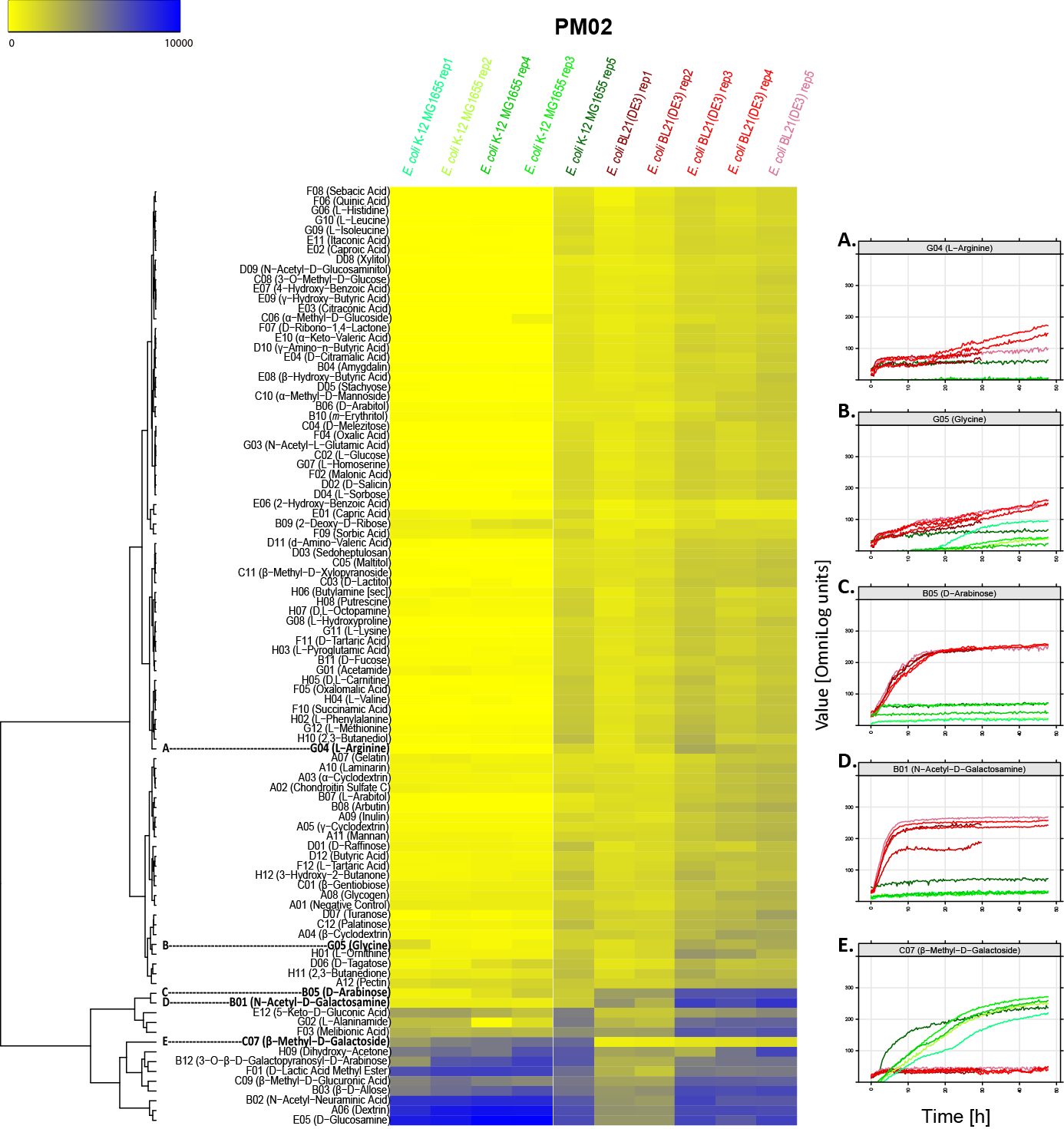

Supplement: S6 Fig — (DOCX) [file pone.0204375.s006.docx]
